# Time for a global response to labour rights violations in the manufacture of health-care goods

**DOI:** 10.2471/BLT.17.193417

**Published:** 2017-05-01

**Authors:** Mahmood F Bhutta

**Affiliations:** a Medical Fair and Ethical Trade Group, International Department, British Medical Association, BMA House, Tavistock Square, London WC1H 9JP, England.

On 23 March 2017, the British Medical Association, in partnership with the Sustainable Development Unit for the health sector in England, released *Ethical procurement for health: workbook 2.0*,^1^ a guide and workbook for protecting labour rights in medical supply chains. This is an update to a document released 6 years earlier in response to evidence of abuse of worker rights at several factories manufacturing health-care products destined for global markets.

Poor labour conditions should concern all those in health care. Work is inextricably correlated to physical and mental well-being: unsafe working conditions risk bodily injury; inadequate remuneration links to malnutrition, poor housing and lack of opportunity.^2^ Long or irregular working hours and a lack of respect at work contribute to stress, anxiety and depression.^3^

Working conditions found in the manufacture of some health-care products have been among the worse encountered anywhere.

Most medical gloves are manufactured in factories in Malaysia or Thailand, where employees are usually immigrant workers.^4^ Interviews with some of these workers revealed that many had secured employment by paying extortionate fees to international recruitment agencies in exchange for poorly paid jobs.^4^ Most will work 12–13 hours a day, 7 days a week, and suffer exhaustion.^4^ Some have been subject to bullying and harassment. In one instance, an employee alleged that he was imprisoned in a factory and threatened with physical violence by the employer.^4^

An estimated two-thirds of the 150 million reusable and disposable surgical instruments in the global market are produced each year in Sialkot in northern Pakistan.^5^ Here, over 50 000 workers do most of the filing, grinding, hammering and polishing of instruments by hand.^5^ They will regularly work over 80 hours a week for an inadequate wage, and frequently suffer musculoskeletal injuries, that are sometimes incapacitating. The surgical instruments’ industry in Pakistan employs hundreds of children, some aged as young as 7 years. Many children work to pay off debt to their father’s employer. Most work full time and receive no formal education and are thus trapped in poverty.

Long working hours and low pay has also been noted in the manufacture of surgical masks in Mexico^6^ and health-care uniforms in India.^7^ People working in the manufacture of medical goods, in general, are considered at high risk of labour rights abuse^8^ because most of the factories are located in countries where such abuse is frequent.

The British Medical Association, through its Medical Fair and Ethical Trade Group,^9^ has coordinated an international response to this issue. Suppliers of goods have been reactive in their need to protect workers. Significant progress has only been made through developing new regional and national procurement policies.

Since 2010, health care procurement in all 21 regions of Sweden has been bound by a code of conduct,^10^ requiring suppliers to comply with the International Labour Organization *Declaration on fundamental principles and rights at work*, as well as with local employment and health and safety legislation. For high-risk products, suppliers are also contractually required to allow independent audit of manufacturing sites to identify problems and to oblige remedial action. This requirement has led to demonstrable improvements in working conditions for the people making surgical instruments,^11^ gloves^4^ and textiles that supply the Swedish health-care system. Monitoring has led to better remuneration, a reduction in working hours, improved health and safety and elimination of unfair recruitment fees in factories in India, Malaysia and Pakistan. A similar approach has been used by South-Eastern Norway Regional Health Authority^12^ with comparable outcomes.

In England, the single largest procurer of medical goods is the National Health Service (NHS) Supply Chain. In 2007, the NHS Supply Chain released a supplier code of conduct, and then in 2012 introduced the Labour Standards Assurance System, a contractual obligation for suppliers of surgical instruments to assess and continually improve working conditions.^13^ This system has now been extended to other high-risk products, including textiles and gloves. This approach has been successful: only one of 29 approved surgical instrument suppliers to the NHS Supply Chain subsequently had their contract terminated for failure to demonstrate protection of worker rights.

These policies are a step in the right direction, leading to hundreds of millions of United States dollars (US$) of health care purchasing coming under scrutiny for labour rights protection. Yet this still represents only a small proportion of the global market for health-care products, which is growing, and estimated to soon be worth over US$ 500 billion annually.^14^

When more health-care providers adopt ethical procurement policies, respect for workers will become a norm in this industry. *Ethical procurement for health: workbook 2.0* is a resource that provides governments, institutions and individuals with guidance and a toolkit to help achieve that aim.
